# Advances in CD30- and PD-1-targeted therapies for classical Hodgkin lymphoma

**DOI:** 10.1186/s13045-018-0601-9

**Published:** 2018-04-23

**Authors:** Yucai Wang, Grzegorz S. Nowakowski, Michael L. Wang, Stephen M. Ansell

**Affiliations:** 1 Division of Hematology, Mayo Clinic, 200 First St SW, Rochester, MN 55905 USA; 2 Department of Lymphoma/Myeloma, The University of Texas MD Anderson Cancer Center, Houston, TX USA

## Abstract

CD30 and programmed cell death protein 1 (PD-1) are two ideal therapeutic targets in classical Hodgkin lymphoma (cHL). The CD30 antibody-drug conjugate (ADC) brentuximab vedotin and the PD-1 antibodies nivolumab and pembrolizumab are highly efficacious in treating relapsed and/or refractory cHL. Ongoing studies are evaluating their efficacy in earlier lines of therapy and have demonstrated encouraging results. These agents are expected to further change the landscape of cHL management. Increased cure rates and reduced long-term toxicity from traditional chemotherapy and radiotherapy are likely with the emergence of these novel targeted therapies.

## Background

Hodgkin lymphoma (HL) accounts for approximately 10% of lymphomas. It is estimated that there will be 8500 new cases of HL and 1050 deaths from this disease in the USA in 2018 [1]. The age at diagnosis shows a bimodal distribution, with one peak between 15 and 34 and the other after 60 [2]. Pathologically, HL is characterized by clonal malignant Hodgkin and Reed-Sternberg (HRS) or variant cells, which reside in an extensively inflammatory tumor microenvironment. Based on morphology and HRS cell phenotypes, HL can be divided into classical HL (cHL) and nodular lymphocyte predominant Hodgkin lymphoma (NLPHL), with the latter accounting for about 5% of Hodgkin lymphoma cases. In cHL, the characteristic HRS cells are giant, mononucleated (Hodgkin cells) or multinucleated (Reed-Sternberg cells), with prominent eosinophilic inclusion-like nucleoli. They are typically positive for CD15 and CD30. The HRS cells produce various cytokines, through which they interact with surrounding immune cells to drive their own proliferation and survival and regulate the host response. In NLPHL, the HRS variant cells are termed lymphocyte-predominant (LP) cells and are typically negative for CD15 and CD30 but positive for CD20. The treatment strategies for cHL and NLPHL differ due to distinct biology.

With standard frontline chemotherapy such as ABVD (adriamycin, bleomycin, vinblastine, dacarbazine) or BEACOPP (bleomycin, etoposide, doxorubicin, cyclophosphamide, vincristine, procarbazine, prednisone) and radiotherapy when indicated, the majority of cHL patients can be cured. However, approximately 10% of early-stage and 30% of advanced stage cHL patients experience disease relapse after frontline therapy or have refractory disease that fails to respond to frontline therapy, and only half of them can be cured with salvage chemotherapy followed by high-dose therapy (HDT) and autologous stem cell transplantation (ASCT) [2]. Until 10 years ago, there was a distinct lack of efficacious treatment options for cHL patients who relapsed after second-line therapy.

**Fig. 1:**
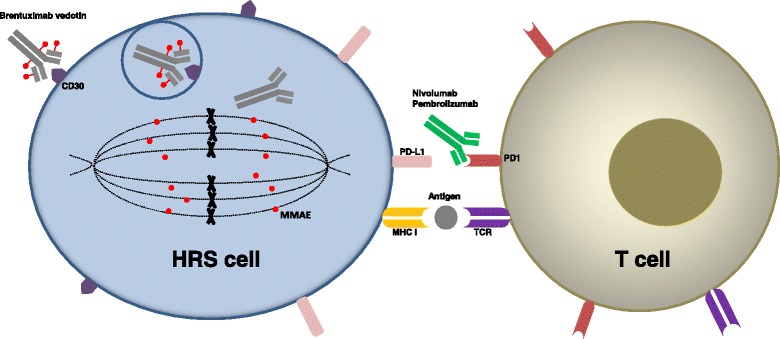
Mechanisms of action of brentuximab vedotin and anti-PD-1 antibodies. Brentuximab vedotin binds to CD30 on the HRS cell surface and gets internalized into the cell via endocytosis. The cytotoxic MMAE then gets cleaved from the anti-CD30 antibody and interrupts mitosis. The anti-PD-1 antibodies nivolumab and pembrolizumab bind to PD-1 on T cells and block the PD-L1/PD-1-mediated immune checkpoint signaling, allowing reactivation of T cells that exert cytotoxic function against HRS cells. HRS, Hodgkin and Reed-Sternberg; MMAE, monomethyl auristatin E; MHC I, major histocompatibility complex (MHC) type I; TCR, T cell receptor

Several novel therapies for cHL have emerged in recent years. The two major targets are CD30, which is expressed by HRS cells, and programmed cell death protein 1 (PD-1), the receptor for programmed death-ligand 1 (PD-L1) and PD-L2 which are almost universally overexpressed in HRS cells due to chromosome 9p24.1 amplification [3]. The CD30 antibody-drug conjugate (ADC) brentuximab vedotin and the PD-1 inhibitors nivolumab and pembrolizumab are approved by the United States Food and Drug Administration (FDA) and have significantly changed the landscape of cHL management. Their mechanisms of action are shown in Fig. 1. Agents targeting the JAK/STAT and PI3K/Akt/mTOR pathways, histone acetyltransferase (HDAC) inhibitors and immunomodulatory drugs (IMiDs) have also been investigated, with some promising results demonstrated in early-phase clinical studies [4]. In this article, we review the advances in CD30- and PD-1-targeted therapies in cHL, with a focus on major completed or ongoing studies on brentuximab vedotin, nivolumab, and pembrolizumab. Of note, although “classical HL” was not explicitly specified in some of these studies, all the data should be on cHL, given the CD30 expression and 9p24.1 amplification in cHL and distinct management approaches of cHL and NLPHL.

## CD30-targeted therapy

### Monoclonal antibodies and mAb-immunotoxin conjugates

CD30 is a member of the tumor necrosis factor (TNF) receptor superfamily. In healthy individuals, its expression is restricted to a small fraction of activated B and T cells. In cHL, CD30 is highly expressed on HRS cells and is an ideal therapeutic target. However, the activity of several unconjugated anti-CD30 monoclonal antibodies (mAbs) in cHL was minimal. In an early study, a CD30 mAb Ber-H2 demonstrated excellent in vivo targeting of HRS cells but did not have an antitumor effect in six patients treated [5]. In a phase 1/2 study, a fully human CD30 mAb MDX-60 showed only limited activity against HL, with 2 complete responses (CR) and 2 partial responses (PR) in 63 patients [6]. In another phase 1 study of the chimeric mAb SGN-30 (cAC10), no objective response was seen in 21 HL patients [7]. In a phase 2 study, SGN-30 did not result in any objective response in HL either [8]. The reason for the poor activity of these unconjugated anti-CD30 mAbs in cHL is unclear. One potential mechanism may be insufficient cell killing following CD30 binding. We suspect that the antibody-dependent cell killing mechanisms such as antibody-dependent cell-mediated cytotoxicity and antibody-dependent cellular phagocytosis are compromised in these heavily pretreated HL patients with immunosuppression.

On the other hand, mAb-immunotoxin conjugates seemed to have some limited activity in HL. In an early study, a mAb-immunotoxin conjugate Ber-H2-saporin resulted in tumor reduction in all four treated patients, although duration of response was limited (up to 10 weeks) [9]. In a phase 1 study, Ki-4.dgA, a conjugate of CD30 mAb and deglycosylated ricin A-chain, produced 1 PR and 1 minor response (MR) in 15 HL patients [10].

### Brentuximab vedotin

#### Therapy for relapsed or refractory disease

The breakthrough in CD30-targeted therapy in HL came with the development of brentuximab vedotin (SGN-35), an ADC consisting of chimeric CD30 mAb brentuximab (cAC10) and an antimitotic agent monomethyl auristatin E (MMAE) (Table 1). In early-phase clinical trials, brentuximab vedotin demonstrated impressive single-agent activity in CD30-positive hematological malignancies including HL and anaplastic large-cell lymphoma (ALCL). In the first phase 1 dose-escalation study with doses ranging from 0.1 to 3.6 mg/kg every 3 weeks, the maximum tolerated dose (MTD) was 1.8 mg/kg. In 42 patients with HL, the objective response rate (ORR) was 36%, with 9 CR and 6 PR. For the 12 patients who were treated at the MTD, the ORR was 50%, with 4 CR (33%) and 2 PR (17%) [11]. In another phase 1 dose-escalation study, brentuximab vedotin was given on days 1, 8, and 15 of each 28-day cycle at doses ranging from 0.4 to 1.4 mg/kg. The MTD was 1.2 mg/kg. In 35 HL patients evaluable for efficacy, the ORR was 54%, with 10 CR and 9 PR. For the 12 patients who were treated at the MTD, the ORR was 58%, with 3 CR (25%) and 4 PR (33%) [12]. In a Japanese phase 1/2 study, five CR (56%) and one PR (11%) were observed in nine HL patients in the phase 2 portion (1.8 mg/kg every 3 weeks), with an ORR of 67% [13].

**Table 1:** Major clinical trials on brentuximab vedotin for treatment of Hodgkin lymphoma

Study	Phase	*N*	Setting	Treatment	Efficacy results
Younes et al. 2010 [11]	1	42	Relapsed or refractory	Bv 0.1 to 3.6 mg/kg Q3W MTD 1.8 mg/kg	All patients: ORR 36% (CR 21%, PR 14%) MTD only (*n* = 12): ORR 50% (CR 33%, PR 17%)
Fanale et al. 2012 [12]	1	38	Relapsed or refractory	Bv 0.4 to 1.4 mg/kg on days 1, 8, and 15 of each 28-day cycle MTD 1.2 mg/kg	All evaluable (*n* = 35): ORR 54% (CR 29%, PR 26%) MTD only (*n* = 12): ORR 58% (CR 25%, PR 33%)
Younes et al. 2012 [14–16]	2	102	Relapsed or refractory after ASCT	Bv 1.8 mg/kg Q3W for up to 16 cycles	ORR 75% (CR 34%, PR 40%) Median DOR 20.5 months (CR patients) 3-year follow-up: Median PFS 9.3 months, median OS 40.5 months 5-year follow-up: 5-year PFS rate 22%, 5-year OS rate 41%
O’Connor et al. 2018 [19]	2	37	Relapsed or refractory	Bv 1.8 mg/kg day 1 and bendamustine 90 mg/m^2^ day 1–2 Q3W for up to 6 cycles	ORR 78% (CR 43%, PR 35%) Median PFS and median OS not reached
Moskowitz et al. 2015 (AETHERA) [21]	3	329	Consolidation after ASCT	Bv 1.8 mg/kg vs placebo Q3W for 16 cycles	Bv (*n* = 165) vs placebo (*n* = 164): Median PFS 42.9 vs 24.1 months (HR = 0.57, *P* = 0.0013)
Chen et al. 2015 [22, 23]	2	37	Relapsed after or refractory to frontline therapy	Bv 1.8 mg/kg Q3W for 4 cycles	ORR 68% (CR 35%, PR 32%) 18 patients directly proceeded to ASCT; 18 patients received additional salvage chemotherapy and 15 proceeded to ASCT 18-month post-transplant PFS rate 73%
Moskowitz et al. 2015 [24]	2	45	Relapsed after or refractory to frontline therapy	Bv 1.2 mg/kg days 1, 8, 15 Q4W for 2 cycles	12 (27%) were PET negative and proceeded to ASCT; 32 received additional salvage chemotherapy and proceeded to ASCT 2-year EFS rate 80%, 2-year OS rate 95%
Cassaday et al. 2017 [25]	1/2	24	Relapsed after or refractory to frontline therapy	Bv 1.2 or 1.5 mg/kg days 1, 8 Q3W in combination with ICE for 2 cycles	20 (87%) of 23 evaluable patients achieved PET CR 19 had undergone ASCT
Garcia-Sanz et al. 2016 [26]	2	66	Relapsed after or refractory to frontline therapy	Bv 1.8 mg/kg Q3W in combination with ESHAP for 3 cycles	Pre-ASCT ORR 96% (CR 70%, PR 26%) 61 had undergone an ASCT Projected 1-year post-transplant PFS rate 87%, OS rate 90%
LaCasce et al. 2015 [27]	1/2	53	Relapsed after or refractory to frontline therapy	Bv 1.8 mg/kg Q3W plus bendamustine 90 mg/m^2^ days 1–2 Q3W for up to 6 cycles	ORR 93% (CR 74%, PR 19%) 37 had undergone ASCT Estimated 12-month PFS rate 80%
Younes et al. 2013 [29, 30]	1	51	Newly diagnosed stage IIA bulky disease or stage IIB–IV	Bv 0.6, 0.9, or 1.2 mg/kg Q2W in combination with ABVD or AVD for up to 6 cycles (28-day)	Bv+ABVD arm (*n* = 25, dose escalation): CR 95% 5-year FFS rate 79%, OS rate 92% Bv+AVD arm (*n* = 26, 1.2 mg/kg only): CR 96% 5-year FFS rate 92%, OS rate 100%
Connors et al. 2018 (ECHELON-1) [31]	3	1334	Untreated stage III or IV	Bv 1.2 mg/kg Q2W in combination with AVD vs ABVD, for up to 6 cycles (28-day)	Bv+AVD vs ABVD: ORR 86 vs 83% CR 73 vs 70% 2-year modified PFS rate 82.1 vs 77.2% (HR = 0.77, *P* = 0.03)
Abramson et al. 2015 [33]	2	34	Newly diagnosed non-bulky stage I–II	Bv 1.2 mg/kg Q2W for 1 cycle (28-day), followed by Bv+AVD for 4–6 cycles (28-day)	After first cycle of Bv: CR 53% After 2 cycles of Bv+AVD: CR 97% At the end of treatment: CR 88% PFS rate 90% and OS rate 97% (median follow-up 14 months)
Kumar et al. 2016 [34]	2	30	Newly diagnosed stage I–II with unfavorable risk factors	Bv 1.2 mg/kg Q2W in combination with AVD for 4 cycles (28-day), followed by 30 Gy ISRT if PET negative	After 2 cycles: 90% PET negative After 4 cycles: 93% PET negative 1-year PFS rate 93.3%
Evens et al. 2017 [37]	2	48	Newly diagnosed stage IIB–IV, age ≥ 60 years	Bv 1.8 mg/kg Q3W for 2 cycles, followed by AVD for 6 cycles, followed by 4 more cycles of Bv if responded	For evaluable patients (*n* = 41) After 2 cycles of Bv: ORR 87% (CR 30%) After completion of AVD: ORR 95% (CR 90%) At end of therapy: ORR 95% (CR 93%) 2-year PFS rate 90%
Park et al. 2016 [38, 39]	2	41	Untreated limited stage non-bulky	ABVD for 2–6 cycles, followed by Bv 1.8 mg/kg Q3W for 6 cycles	After 2 cycles of ABVD: 72% PET negative After completion of Bv: 90% PET negative Estimated 2-year PFS rate 92%, OS rate 97%
Federico et al. 2016 [40]	2	12	Untreated stages IA, IIA, and IIIA	Bv 1.8 mg/kg for 2 cycles, followed by ABVD for 3 or 6 cycles, and radiation therapy if indicated	After 2 cycles of Bv: ORR 92% (CR 83%, PR 8%) At the end of therapy: ORR 100% (CR 92%, PR 8%) 1-year PFS rate 92%
Eichenauer et al. 2017 [41]	2	104	Newly diagnosed advanced stage	Bv 1.2 mg/kg plus ECAPP or ECADD for 6 cycles (21-day)	Bv+ECAPP arm (49 evaluable): CR 86%, 18-month PFS rate 95% Bv+ECADD arm (52 evaluable): CR 88%, 18-month PFS rate 89%
Friedberg et al. 2017 [42]	2	42	Treat-naïve, age ≥ 60 years, ineligible for or declined standard frontline chemotherapies	Bv 1.8 mg/kg plus dacarbazine 375 mg/m^2^ Q3W for 12 cycles followed by Bv 1.8 mg/kg for 4 cycles or more, or Bv 1.8 mg/kg day 1 plus bendamustine 90 mg/m^2^ days 1–2 Q3W for 6 cycles followed by Bv 1.8 mg/kg for 10 cycles or more	Bv+dacarbazine arm (21 evaluable): ORR 100% (CR 62%, PR 38%) Median PFS 17.9 months Bv + bendamustine arm (20 evaluable): ORR 100% (CR 88%, PR 12%) Median PFS not reached
Forero-Torres et al. 2015 [44]	2	27	Treat-naïve, age ≥ 60 years, ineligible for or declined conventional combination treatment	Bv 1.8 mg/kg Q3W for up to 16 cycles; additional cycles allowed in those with clinical benefit	ORR 92% (CR 73%, PR 19%) Median PFS 10.5 months
Gibbs et al. 2017 (BREVITY) [45]	2	38	Untreated, unfit for standard treatment	Bv 1.8 mg/kg Q3W for up to 16 cycles	For evaluable patients (*n* = 31): CMR after 4 cycles 26% ORR 84% Median PFS 7.4 months

*N* patient number; *Bv* brentuximab vedotin; *Q3W* every 3 weeks; *Q4W* every 4 weeks; *MTD* maximum tolerated dose; *ABVD* adriamycin, bleomycin, vinblastine, dacarbazine; *AVD* adriamycin, vinblastine, dacarbazine; *ECADD* etoposide, cyclophosphamide, doxorubicin, dacarbazine, and dexamethasone; *ECAPP* etoposide, cyclophosphamide, doxorubicin, procarbazine, and prednisone; *ESHAP* etoposide, Solu-Medrol, high-dose cytarabine, cisplatin; *ICE* ifosfamide, carboplatin, etoposide; *ISRT* involved-site radiotherapy; *ASCT* autologous stem cell transplantation; *ORR* objective response rate; *CR* complete response; *PR* partial response; *CMR* complete metabolic response; *PFS* progression-free survival; *FFS* failure-free survival; *OS* overall survival; *HR* hazard ratio

In the pivotal phase 2 trial of brentuximab vedotin in relapsed or refractory HL after ASCT, 102 patients were treated with brentuximab vedotin (1.8 mg/kg) every 3 weeks for a maximum of 16 cycles. The most common treatment-related adverse events (AEs) were peripheral sensory neuropathy, nausea, fatigue, neutropenia, and diarrhea. The ORR was 75%, with 34% CR and 40% PR. The median duration of response (DOR) in those with a CR was 20.5 months [14]. At 3-year follow-up, median progression-free survival (PFS) and median overall survival (OS) was 9.3 and 40.5 months, respectively [15]. With longer follow-up, the 5-year PFS and OS rates were 22 and 41%, respectively. In patients who achieved CR, the 5-year PFS and OS rates were 52 and 64%, respectively, and the median PFS and OS were not reached suggesting that some patients may be cured [16]. This pivotal trial met its primary endpoint (ORR) and was the basis for FDA approval of brentuximab vedotin in August 2011 for patients with HL after failure of ASCT or at least two prior multi-agent chemotherapy regimens if not eligible for ASCT. This was the first FDA-approved treatment for HL since 1977.

Brentuximab vedotin also demonstrated activity in relapsed or refractory HL after allogeneic stem cell transplantation (Allo-SCT). In a multicenter study, 25 patients received 1.2 or 1.8 mg/kg of brentuximab vedotin every 3 weeks. The ORR was 50%, with 38% CR, and the median PFS was 7.8 months [17].

In patients with relapsed or refractory HL previously treated with brentuximab vedotin, retreatment may be an option. This is supported by a phase 2 study in HL and ALCL patients with disease progression or relapse following discontinuation of brentuximab vedotin after achieving CR or PR (on a previous clinical trial). In 20 HL patients evaluable for efficacy, the ORR was 60%, with 30% CR and 30% PR. The median DOR was 9.2 months, and the median PFS was 9.9 months. AE profile during retreatment was similar to that observed in the pivotal trials, with the exception of higher rates of peripheral neuropathy due to the cumulative effect [18].

Brentuximab vedotin in combination with bendamustine was tested in relapsed or refractory HL. In a phase 1/2 study, patients (predominantly HL) were treated with brentuximab vedotin on day 1 and bendamustine on days 1 and 2 of every 3-week cycle for up to 6 cycles. Forty-two patients had received an ASCT and three patients an Allo-SCT prior to enrollment. Eleven patients were previously exposed to brentuximab vedotin. In the phase 1 portion of the study, the MTD was not reached. The highest dose (brentuximab vedotin 1.8 mg/kg and bendamustine 90 mg/m^2^) was chosen for phase 2. The ORR in phase 1 (*n* = 28, 27 HL) was 61%, with 18% CR and 43% PR. The ORR in phase 2 (*n* = 37, all HL) was 78%, with 43% CR and 35% PR. The median PFS and OS were not reached in phase 2 [19]. Although the ORR was similar, the CR rate appeared higher compared to that in the pivotal phase 2 study of single-agent brentuximab vedotin [14]. Higher dose of bendamustine may further improve the efficacy of this combination. In a single-center cohort study presented at the 2017 annual meeting of American Society of Hematology (ASH 2017), the combination of brentuximab vedotin (1.8 mg/kg, day 3) and bendamustine (120 mg/m^2^, day 1–2) for 4–6 28-day cycles resulted in 100% CR in 11 patients with relapsed or refractory HL after ASCT. Six patients then underwent a second ASCT, and five patients underwent haploidentical transplant. All patients remained in CR after a median follow-up of 33.4 months [20].

#### Consolidation therapy after ASCT

The AETHERA trial is a randomized, placebo-controlled phase 3 study that assessed whether brentuximab vedotin could improve PFS when given as early consolidation after ASCT in cHL patients with a high relapse risk (primary refractory, relapse within 12 months, extranodal involvement at the start of pre-transplantation salvage chemotherapy) [21]. Brentuximab vedotin (1.8 mg/kg) or placebo was administered every 3 weeks for 16 cycles, starting 30–45 days after transplantation. The median PFS was significantly improved in patients in the brentuximab vedotin arm (42.9 vs 24.1 months, hazard ratio (HR) = 0.57, *P* = 0.0013). Based on the substantial difference in PFS, National Comprehensive Cancer Network (NCCN) currently recommends 1 year of brentuximab vedotin consolidation therapy following ASCT in cHL patients with a high risk of relapse.

#### Salvage therapy after frontline therapy

The role of brentuximab vedotin in salvage therapy for patients with refractory or relapsed disease after frontline therapy was evaluated by a few studies. In a phase 2 study led by the City of Hope investigators, cHL patients were treated with brentuximab vedotin (1.8 mg/kg every 3 weeks) for a total of 4 cycles and proceeded to ASCT if eligible with or without additional salvage therapy based on remission status. The ORR in 37 treated patients was 68%, with 35% CR and 32% PR [22]. Eighteen patients (13 CR, 4 PR, 1 stable disease (SD; received additional radiation therapy) proceeded to ASCT without additional salvage chemotherapy. Eighteen patients received additional salvage therapy, with 11 achieving CR and 4 achieving PR and proceeded to ASCT [22, 23]. After a median follow-up of 17.6 months, the 18-month post-transplant PFS rate was 73% [23]. In another phase 2 study conducted at the Memorial Sloan Kettering Cancer Center, 45 patients were treated with brentuximab vedotin (1.2 mg/kg on days 1, 8, and 15 of every 4-week cycle) for 2 cycles. Twelve patients (27%) achieved positron emission tomography (PET) negativity and directly proceeded to ASCT. Thirty-two of the 33 PET-positive patients went on to receive 2 cycles of augmented ICE (ifosfamide, carboplatin, etoposide). Twenty-two of them achieved PET negativity and proceeded to ASCT. The other 10 patients all went on to ASCT eventually (1 after 1 additional cycle of augmented ICE, 6 after involved-field radiation therapy (IFRT), and 3 without additional therapy). After a median follow-up of 20.1 months, the 2-year event-free survival (EFS) was 80% and OS was 95% [24]. These results demonstrated that brentuximab vedotin is an active first-line salvage therapy, producing 27–35% CR as a single agent. Another 30–49% of patients could achieve a CR with additional salvage therapy. The majority of patients could proceed to ASCT.

Brentuximab vedotin in combination with conventional salvage chemotherapy can potentially improve outcome with salvage therapy and make more cHL patients eligible for ASCT. The brentuximab vedotin and ICE combination produced a high PET CR rate as first-line salvage therapy in an ongoing phase 1/2 trial at the University of Washington. Brentuximab vedotin (1.2 or 1.5 mg/kg in phase 1 dose escalation, and 1.5 mg/kg in phase 2) was given on days 1 and 8 every 3 weeks in combination with ICE for 2 cycles. Therapy was well tolerated. At the time of report at ASH 2017, 20 (87%) of 23 evaluable cHL patients achieved PET CR and 19 had undergone an ASCT [25]. The combination of brentuximab vedotin with ESHAP (etoposide, Solu-Medrol, high-dose cytarabine, cisplatin) is also highly effective as a first-line salvage therapy. In a GELTAMO phase 2 trial, patients were treated with brentuximab vedotin (1.8 mg/kg every 3 weeks) and ESHAP for 3 cycles. At the time of report at ASH 2016, 66 cHL patients completed pre-transplant treatment. The addition of brentuximab vedotin did not result in a higher toxicity. The ORR was 96%, with 70% CR and 26% PR. Sixty-one patients had undergone ASCT. In 47 patients with available data, 37 (80%) were in CR and 3 (7%) were in PR after transplant. With a mean follow-up of 11 months, the projected 1-year post-transplant PFS rate was 87% and OS rate was 90% [26].

Brentuximab vedotin in combination with bendamustine was also very effective for first-line salvage therapy. In a phase 1/2 study, brentuximab vedotin (1.8 mg/kg every 3 weeks) and bendamustine (90 mg/m^2^ on days 1 and 2 every 3 weeks) for up to 6 cycles resulted in a CR rate of 74% (39/53) and an ORR of 93% (49/53) [27]. The response rates were higher compared to those when the combination was used in multiply relapsed or refractory patients [19]. Thirty-seven patients went on to ASCT. The estimated 12-month PFS was 80% for both the transplanted population and the overall population [27]. In a phase 2 trial by the Children’s Oncology Group, pediatric and young adult patients (≤ 30 years) were treated with brentuximab vedotin (1.8 mg/kg every 3 weeks) and gemcitabine (1000 mg/m^2^ on days 1 and 8 every 3 weeks) for up to 4 cycles, and the CR rate was 58% (23/40) and the ORR was 73% (29/40) [28]. While the combination of brentuximab vedotin and gemcitabine appeared less effective in this setting, one should note that the populations in the studies were very different.

#### Frontline therapy

Incorporation of brentuximab vedotin in frontline therapy of HL is a current research focus, in the hope of improving cure rates. In the initial phase 1 dose-escalation study, cHL patients with stage IIA bulky disease or stage IIB-IV disease were treated with brentuximab vedotin plus ABVD or adriamycin, vinblastine, dacarbazine (AVD) for up to 6 cycles. The MTD of brentuximab vedotin in this setting was not exceeded at 1.2 mg/kg. The CR rate was 95% (21/22) in the ABVD arm and 96% (24/25) in the AVD arm [29]. With long-term follow-up, the 5-year failure-free survival (FFS) and OS rates were 79 and 92% in the brentuximab vedotin plus ABVD arm and 92 and 100% in the brentuximab vedotin plus AVD arm, respectively [30]. Importantly, the brentuximab vedotin plus ABVD combination produced a high rate of pulmonary toxicity (44%) [29], suggesting intolerance and infeasibility for further development. Following this study, the large phase 3 ECHELON-1 study compared brentuximab vedotin plus AVD (*n* = 664) with ABVD (*n* = 670) in previously untreated stage III or IV cHL. Brentuximab vedotin (1.2 mg/kg) plus AVD or ABVD were given on days 1 and 15 every 4 weeks for up to 6 cycles. The primary end point was modified PFS, defined as time to disease progression, death, or modified progression (evidence of non-complete response after completion of frontline therapy, followed by subsequent anticancer therapy). The brentuximab vedotin plus AVD regimen resulted in more myelosuppression and neurotoxicity but substantially less pulmonary toxicity and appeared more effective for frontline treatment for advanced stage cHL. The CR and ORR rates were 73 and 86% in the brentuximab vedotin plus AVD group and 70 and 83% in the ABVD group. After a median follow-up of 24.9 months, the 2-year modified PFS rate was significantly higher in the brentuximab vedotin plus AVD group (82.1 vs 77.2%, HR = 0.77, *P* = 0.03) [31]. Based on these data, brentuximab vedotin was approved by FDA in March 2018 to treat adult patients with previously untreated stage III or IV cHL in combination with chemotherapy. Brentuximab vedotin plus AVD will potentially challenge the ABVD regimen as the new standard therapy for advanced stage cHL patients. However, the need for growth factor support and the high cost associated with this regimen must be considered. In addition, since the RATHL trial showed equivalent outcomes for cHL patients treated with 6 cycles of ABVD versus 2 cycles of ABVD followed by 4 cycles of AVD in the setting of a negative interim PET [32], the advantage of reduced pulmonary toxicity of brentuximab vedotin plus AVD compared to ABVD for 6 cycles in the ECHELON-1 trial will likely be less appealing.

In early-stage HL, brentuximab vedotin in combination with AVD have been studied in a few trials, and it is highly effective. In a phase 2 study, cHL patients with non-bulky stage I–II disease received a lead-in cycle of brentuximab vedotin (1.2 mg/kg, days 1 and 15), followed by brentuximab vedotin plus AVD for 4–6 cycles. After the monotherapy lead-in, 18 of 34 (53%) patients achieved CR. After 2 cycles of brentuximab vedotin plus AVD, 33 patients (97%) were in CR (1 off study due to toxicity). At the end of treatment, 30 (88%) patients were in CR (2 had progressive disease (PD) and 2 were off study due to toxicity). After a median follow-up of 14 months, PFS and OS rates were 90 and 97%, respectively [33]. In a phase 2 study of stage I–II cHL with unfavorable risk factors, brentuximab vedotin (1.2 mg/kg) and AVD were given for 4 cycles (days 1 and 15 of the 28-day cycle), followed by 30 Gy involved-site radiation therapy (ISRT) if PET was negative. After 2 and 4 cycles of therapy, 90% (26/29) and 93% (27/29) of patients were PET negative, respectively. All 25 patients who completed chemotherapy and ISRT achieved CR. After a median follow-up of 18.8 months, the 1-year PFS rate by intention to treat (ITT) was 93.3% [34]. In the second cohort of this study, ISRT was reduced to 20 Gy. The response rates were identical, with 90% (26/29) and 93% (27/29) of patients being PET negative after 2 and 4 cycles of therapy, respectively. All 28 patients who completed all therapies achieved CR [35]. Finally, an ongoing randomized phase 2 trial by the Lysa-FIL-EORTC intergroup is comparing 4 cycles of brentuximab vedotin plus AVD with ABVD followed by 30 Gy of involved-node radiation therapy (INRT) in stage I–II HL with unfavorable risk factors. At the time of report at ASH 2017, 93 of 113 patients (82.3%) in the brentuximab vedotin plus AVD arm and 43 of 57 patients (75.4%) in the ABVD arm were PET negative after 2 cycles of treatment [36].

For older patients with advanced stage disease, a sequential brentuximab vedotin and AVD regimen was investigated in a multicenter phase 2 study. Patients ≥ 60 years with stage IIB–IV cHL were treated with two lead-in cycles of brentuximab vedotin (1.8 mg/kg every 3 weeks) followed by 6 cycles of AVD. Responding patients went on to receive four more cycles of consolidative brentuximab vedotin. In 41 evaluable patients, the CR and ORR rates after the lead-in were 30 and 87%, respectively. After completion of AVD, the CR and ORR rates were 90 and 95%, respectively. One patient has improved response to CR after brentuximab vedotin consolidation, resulting in a final CR rate of 93%. After a medium follow-up of 24 months, the 2-year PFS rate was 90% [37].

Given the excessive pulmonary toxicity, combination of brentuximab vedotin and ABVD is not feasible for further investigation [29]. However, sequential therapy has been investigated in a couple of studies. In a multicenter phase 2 study, patients with limited stage non-bulky HL were treated with ABVD for 2–6 cycles, followed by brentuximab vedotin (1.8 mg/kg every 3 weeks) for 6 cycles. After 2 cycles of ABVD, 72% of the 40 evaluable patients were PET negative. After completion of brentuximab vedotin consolidation, 90% of patients were PET negative. With a median follow-up of 12 months, the estimated 1-year PFS and OS rates were 91 and 96%, respectively [38]. The updated results showed that 37 of 39 evaluable patients were PET negative after the completion of therapy. With a median follow-up of 22 months, the estimated 2-year PFS and OS rates were 92 and 97%, respectively [39]. In another phase 2 study, patients with stage IA, IIA, and IIIA HL were treated with brentuximab vedotin (1.8 mg/kg) for 2 cycles followed by ABVD for 3 or 6 cycles, with or without radiation therapy. After 2 cycles of brentuximab vedotin, 10 of 12 patients (83%) achieved CR and 1 patient (8%) achieved PR. At the end of therapy, the ORR was 100%, with 92% CR and 8% PR. The 1-year PFS rate was 92% [40].

The German Hodgkin lymphoma Study Group incorporated brentuximab vedotin into modified escalated BEACOPP as frontline therapy for advanced stage cHL. Patients were randomized to 6 cycles of either brentuximab vedotin plus ECAPP (etoposide, cyclophosphamide, doxorubicin, procarbazine, and prednisone) or brentuximab vedotin plus ECADD (etoposide, cyclophosphamide, doxorubicin, dacarbazine, and dexamethasone). After completion of chemotherapy, the CR rates were 86% (42/49) in the BrECAPP (brentuximab vedotin, etoposide, cyclophosphamide, doxorubicin, procarbazine, and prednisone) arm and 88% (46/52) in the BrECADD (brentuximab vedotin, etoposide, cyclophosphamide, doxorubicin, dacarbazine, and dexamethasone) arm. Consolidation radiotherapy was given to seven patients in the BrECAPP arm and six patients in the BrECADD arm (Deauville score of 4 or above). After completion of all therapies, the CR rate increased to 94% (46/49) in the BrECAPP arm but remained at 88% (46/52) in the BrECADD arm. The 18-month PFS rate was 95% in the BrECAPP arm and 89% in the BrECADD arm [41]. The brentuximab vedotin plus ECADD regimen was associated with a more favorable toxicity profile and is currently being compared to the standard escalated BEACOPP in the phase 3 HD21 study.

Older HL patients tend to do poorly with conventional frontline chemotherapy regimens due to comorbidities and treatment-related toxicities. Less intensive combination regimens have been investigated in elderly patients with HL. In a multicenter phase 2 study, cHL patients ≥ 60 who were ineligible or declined standard frontline therapy such as ABVD or BEACOPP were treated with brentuximab vedotin (1.8 mg/kg) plus dacarbazine (up to 12 cycles) or bendamustine (up to 6 cycles). Additional cycles of brentuximab vedotin were allowed to complete a total of 16 cycles or more. The ORR was 100% in both arms. The CR rate was 62% in the dacarbazine arm (*n* = 22) and 88% in the bendamustine arm (*n* = 20). Although brentuximab vedotin plus bendamustine appeared more active, this regimen also caused more serious AEs (65 vs 18%) [42]. Of note, half of the patients in this study had at least three comorbidities or were impaired in at least one aspect that significantly interfered with their quality of life. In these patients, brentuximab vedotin plus bendamustine should be used with caution and many elderly patients may not be eligible for this therapy at these doses. In the phase 1/2 HALO study in France and Italy, a lower dose of brentuximab vedotin (1.2 mg/kg) plus bendamustine (up to 6 cycles) was used for elderly patients with stage IIB–IVB cHL disease. Twelve patients were treated in phase 1, and the treatment-related toxicities were largely hematological and manageable. The study is currently in the phase 2 portion. At the time of report at ASH 2016, nine patients had PET scans available for efficacy evaluation. All patients achieved complete metabolic response (CMR), seven after 2 cycles and two after 4 cycles [43]. Therefore, a lower dose of brentuximab vedotin in combination with bendamustine can be a promising frontline treatment option for elderly patients.

Single-agent brentuximab vedotin for frontline therapy in elderly HL patients has also been investigated. A multicenter phase 2 clinical trial evaluated the efficacy of brentuximab vedotin in cHL patients 60 years or older. Twenty-seven patients were treated with brentuximab vedotin (1.8 mg/kg every 3 weeks) for up to 16 cycles. Patients with clinical benefit could continue treatment beyond 16 cycles until disease progression, unacceptable toxicity, or study closure. The ORR was 92%, with 73% CR. The median PFS was relatively short at 10.5 months, and the median OS was not reached [44]. The BREVITY trial in UK is another study that evaluated single-agent brentuximab vedotin in newly diagnosed HL patients unsuitable for standard chemotherapy. Thirty-eight patients were enrolled and treated with brentuximab vedotin (1.8 mg/kg) every 3 weeks for up to 16 cycles. At the time of report at the International Conference on Malignant Lymphoma (ICML) in 2017, 31 patients were evaluable for response. CMR rate was 26% after 4 cycles of therapy. The ORR was 84%. The median PFS was 7.4 months [45]. In elderly or unfit patients, brentuximab vedotin produced a high ORR, but the PFS was short. Management of this patient population therefore remains challenging.

### Bispecific antibodies

Immunotherapy with bispecific mAbs that bridge tumor cells and immune effector cells has achieved some success in both solid tumors and hematological malignancies. Effective therapies include catumaxomab for EpCam-positive gastric and ovarian cancer and blinatumomab for CD19-positive acute lymphoblastic leukemia (ALL). In HL, this strategy has been under development. In the 1990s, Hartmann and colleagues developed a murine bispecific Ab, HRS-3/A9, that targets CD30 and CD16 (Fcγ-receptor III on NK cells and mononuclear phagocytes). In the first phase 1/2 trial, they treated 15 patients with relapsed or refractory HL with HRS-3/A9 (four infusions every 3–4 days) and observed 1 CR (lasting 6 months), 1 PR (lasting 3 months), and 3 MR (lasting 1 to 15 months). The treatment was well tolerated and the MTD was not reached at 64 mg/m^2^. However, eight patients developed human anti-mouse immunoglobulin antibodies, and retreatment was prevented by allergic reactions in all five attempted cases [46]. In a subsequent trial, they treated 16 patients with 100 mg HRS-3/A9 (25 mg daily every other day or continuous infusion over 4 days). In cases with an objective response, retreatment was attempted after 4 weeks, and in cases with stable disease, a second course was given after pre-stimulation with IL-2 followed by GM-CSF. Overall, one CR and three PR lasting 5–9 months were observed. The one CR and one of the three PRs were achieved after the second course of HRS-3/A9 with IL-2 pretreatment [47]. Although a second course treatment was uneventful in all eight attempted cases, a third treatment course was attempted in only one patient with considerable urticaria developed, suggesting that repeated treatment is unlikely to be feasible likely due to development of anti-mouse antibodies.

A chimeric bispecific mAb AFM13 targeting CD30 and CD16A was developed recently and appears promising. In a German phase 1 study, 28 patients with heavily pretreated relapsed or refractory cHL received AFM13 at escalating doses (0.01 to 7 mg/kg, weekly for four doses). One more course of treatment could be given if patients achieve SD or better. Therapy was well tolerated, and the MTD was not reached. In 26 evaluable patients, there were 3 PR and 13 SDs. All three patients with PR received a dose of 1.5 mg/kg or higher [48]. The safety profile and efficacy results were encouraging, and the investigators suggested further development with higher doses and prolonged treatment.

Another bispecific mAb H22xKi-4 was developed in the late 1990s, targeting CD30 and CD64 (Fcγ-receptor I on macrophages and monocytes). In a phase 1 study, 10 patients with refractory HL were treated with escalating doses (1–20 mg/m^2^/day every other day for four doses). The therapy was well tolerated. Overall, there were one CR, three PR, and four SDs, indicating encouraging efficacy [49]. However, no further development of CD30/CD64-targeting was reported.

### CD30 CAR-T cells

T cells engineered to express chimeric antigen receptors (CAR) targeting CD19 or CD20 have shown impressive activity in a number of hematologic malignancies such as ALL and B cell lymphoma. CD30 is an attractive target for CAR-T cell-based immunotherapy in HL. Two reports have been published recently using CD30 CAR-T cells. A phase 1 trial conducted in China enrolled 18 patients with relapsed or refractory HL. Patients received an infusion with a mean of 1.56 × 10^7^ CAR-T cells/kg. The therapy was well tolerated, with grade ≥ 3 toxicities occurring in only 2 of 18 patients (liver function test abnormality and left ventricular systolic dysfunction, respectively). Seven patients (39%) achieved PR, and six achieved SD, suggesting promising activity of CD30 CAR-T cells in this heavily pretreated population [50]. Most recently, Baylor investigators reported a phase 1 study of CD30 CAR-T cells in seven HL and two ALCL patients. Patients received one to four infusions of 0.2–2 × 10^8^ CD30 CAR-T cells/m^2^ without conditioning and tolerated the therapy well. Four HL patients received 2 × 10^8^ CD30 CAR-T cells/m^2^. One patient achieved and remained in CR after 2 years following CAR-T cell infusion. Another patient achieved and remained in CR after 2.5 years after two infusions [51]. The durable CR is particularly encouraging, and tumor debulking and lymphodepletion before CD30 CAR-T cell infusion may further enhance their clinical activity.

## PD-1-targeted immunotherapy

It has long been recognized that HRS cells are surrounded by an extensive background of inflammatory and immune cells. However, there is little evidence of an effective antitumor immune response. In fact, the HRS cells produce molecules that inhibit T cell-mediated immune responses. Importantly, PD-L1 expressed on HRS cells engages PD-1 expressed on T cells, induces immune checkpoint inhibition, and causes T cell exhaustion. Shipp and colleagues demonstrated that chromosome 9p24.1 amplification is a recurrent genetic abnormality in the nodular sclerosis type of cHL, leading to overexpression of genes contained in this region including *PD-L1*, *PD-L2*, and *JAK2*. The enhanced JAK-STAT signaling may further increase PD-L1 expression [3]. This inherent genetic abnormality suggests that HL may have genetically determined vulnerability to immunotherapy with PD-1 blockade. Two PD-1 mAbs, nivolumab and pembrolizumab, have demonstrated high activities in the treatment of HL (Table 2).

**Table 2:** Major clinical trials on nivolumab and pembrolizumab for treatment of Hodgkin lymphoma

Study	Trial name	Phase	*N*	Setting	Treatment	Efficacy results
Ansell et al. 2015 [52]	CheckMate 039 (arm 1, expansion cohort)	1	23	Relapsed or refractory	Nivolumab 3 mg/kg Q2W for up to 2 years	ORR 87% (CR 17%, PR 70%) PFS rate at 24 weeks 86%
Younes et al. 2016 [54–56]	CheckMate 205 (cohort B)	2	80	Relapsed or refractory after ASCT and brentuximab vedotin	Nivolumab 3 mg/kg Q2W	ORR 68% (CR 13%, PR 55%) 12-month PFS rate 54.6%, OS rate 94.9% Median PFS 14.7 months
Armand et al. 2018 [56]	CheckMate 205 (cohorts A and C)	2	Cohort A: 63 Cohort C: 100	Cohort A: relapsed or refractory, brentuximab vedotin naïve Cohort C: relapsed or refractory after brentuximab vedotin	Nivolumab 3 mg/kg Q2W	Cohort A: ORR 65% (CR 29%) Median DOR 20.3 months Median PFS 18.3 months Cohort C: ORR 73% (CR 12%) Median DOR 14.5 months Median PFS 11.9 months
Herbaux et al. 2017 [58]			20	Relapsed after Allo-SCT	Nivolumab 3 mg/kg Q2W	ORR 95% (CR 42%, PR 52%) 1-year PFS rate 58.2%, OS rate 78.7%
Ramchandren et al. 2017 [59]	CheckMate 205 (cohort D)	2	51	Newly diagnosed advanced stage	Nivolumab 240 mg biweekly for 4 doses, followed by nivolumab plus AVD for 6 cycles	ORR 84% (CR 80%, PR 4%) Modified PFS rate at 9 months 94%
Ansell et al. 2016 [60]	CheckMate 039 (arm 2)	1	31	Relapsed or refractory	Nivolumab 3 mg/kg plus ipilimumab 1 mg/kg Q3W for 4 cycles, followed by nivolumab 3 mg/kg Q2W for up to 2 years	ORR 74% (CR 19%, PR 55%)
Herrera et al. 2017 [62]		1/2	62	Relapsed after or refractory to frontline therapy	Nivolumab 3 mg/kg plus brentuximab vedotin 1.8 mg/kg Q3W for up to 4 cycles	ORR 83% (CR 62%)
Armand et al. 2016 [63, 64]	KEYNOTE-013	1b	31	Relapsed or refractory after brentuximab vedotin	Pembrolizumab 10 mg/kg Q2W for up to 2 years	ORR 65% (CR 16%, PR 48%) median PFS 11.4 months 6-month PFS rate 66%, OS rate 100% 12-month PFS rate 48%, OS rate 87%
Chen et al. 2017 [65]	KEYNOTE-087	2	210	Relapsed or refractory after ASCT and/or brentuximab vedotin	Pembrolizumab 200 mg Q3W for up to 2 years	ORR 69.0% (CR 22.4%, PR 46.7%) 6-month PFS rate 72.4%, OS rate 99.5% 9-month PFS rate 63.4%, OS rate 97.5%

*N* patient number, *Q2W* every 2 weeks, *Q3W* every 3 weeks, *AVD* adriamycin, vinblastine, dacarbazine, *ASCT* autologous stem cell transplantation, *ORR* objective response rate, *CR* complete response, *PR* partial response, *PFS* progression-free survival, *OS* overall survival

### Nivolumab

#### Therapy for relapsed and refractory disease

Nivolumab is an IgG4 fully human anti-PD-1 mAb that is efficacious in treating a number of solid tumors. Its activity in HL was first demonstrated in a phase 1 study (CheckMate 039) led by Ansell and colleagues. In the expansion cohort of this study, patients with relapsed or refractory cHL were treated with nivolumab (3 mg/kg) every 2 weeks until disease progression, excessive toxicity, CR, or a maximum of 2 years. At the time of report, 23 patients were enrolled, 78% had received ASCT and 78% had received prior brentuximab vedotin. The ORR was 87%, with 17% CR and 70% PR, and the PFS rate at 24 weeks was 86% [52]. Extended follow-up showed that 10 of the 20 objective responses were durable. In the remaining 10 responders, 5 proceeded to stem cell transplant (4 Allo-SCT and 1 ASCT), 1 discontinued due to toxicity but did not progress at 4-month follow-up, and the other 4 progressed. These data suggest that nivolumab is highly effective in treating multiply relapsed cHL patients, providing durable remissions and/or allowing patients to proceed to a stem cell transplant [53]. Following the CheckMate 039 study, the phase 2 CheckMate 205 study was designed to further evaluate the efficacy of nivolumab in cHL after ASCT. Cohort B of this study was for patients who failed both ASCT and subsequent brentuximab vedotin. Eighty patients were treated with nivolumab (3 mg/kg) every 2 weeks until disease progression, death, or unacceptable toxicity. The ORR was 66%, with 9% CR and 58% PR [54]. According to updated data presented at ASH 2016 based on longer follow-up, the median PFS was 14.8 months. The 12-month PFS rate was 54.6%, and the 12-month OS rate was 94.9%. These data suggest that nivolumab monotherapy can produce durable remissions in heavily pretreated cHL patients after failure of ASCT and brentuximab vedotin [55]. Data from CheckMate 039 and CheckMate 205 were the basis of the FDA approval in May 2016 of nivolumab for cHL that has relapsed or progressed after ASCT and posttransplantation brentuximab vedotin. This was the first approval of checkpoint inhibitors in hematological malignancies.

The activity of nivolumab in treating relapsed and refractory disease is further supported by additional studies. The CheckMate 205 trial also included cHL patients who were brentuximab vedotin naive (cohort A, *n* = 63) and who received brentuximab vedotin before or after ASCT (cohort C, *n* = 100). Patients received the same treatment as those in cohort B. Results after an extended follow-up showed that the ORR in cohorts A, B, and C were 65, 68, and 73%, respectively. The CR rates were 29, 13, and 12%, respectively, and the median DOR was 20.3, 15.9, and 14.5 months in cohorts A–C, respectively. The median PFS in the three cohorts were 18.3, 14.7, and 11.9 months, respectively [56]. These data demonstrated high response rates and durable response to nivolumab regardless of brentuximab vedotin exposure. A Japanese phase 2 study evaluated the efficacy of nivolumab in patients with relapsed or refractory cHL who failed or were ineligible for brentuximab vedotin. Nivolumab (3 mg/kg) was administered every 2 weeks. In 16 patients evaluable for efficacy, the ORR was 81%, with 25% CR and 56% PR. The 6-month PFS and OS rates were 60 and 100%, respectively [57].

A French retrospective study assessed the safety and efficacy of nivolumab in HL patients who relapsed after Allo-SCT. Six (30%) of the 20 treated patients developed graft-versus-host disease (GVHD), all of which had prior history of acute GVHD. Two patients responded to steroid; two patients required alternative immunosuppressive therapy, and two patients died of GVHD. After a median follow-up of 1 year, the 1-year PFS and OS rates were 58.2 and 78.7%, respectively. Thirteen patients were still in response [58]. These data suggest that nivolumab is an effective therapy for this population, although the risk of GVHD is significant.

#### Frontline therapy

Given the efficacy of nivolumab for relapsed and refractory HL, studies are now ongoing investigating its role in frontline therapy of HL. Cohort D of the CheckMate 205 study enrolled patients with newly diagnosed advanced stage cHL (stage II with B symptoms and extra nodal or bulky disease, III and IV). Patients were treated with nivolumab (240 mg biweekly) for four doses, followed by nivolumab plus AVD for 6 cycles. At the time of report at ASH 2017, 51 patients were enrolled, with 49 completed nivolumab monotherapy and 44 completed nivolumab plus AVD. At the end of therapy, the ORR per investigator review for the ITT population was 84%, with 80% CR and 4% PR. The modified PFS rate at 9 months was 94% [59]. The ORR and CR rates were at least similar to those for the brentuximab vedotin plus AVD and ABVD arms in the ECHELON-1 study, suggesting that nivolumab plus AVD is another promising frontline therapy for cHL, although longer follow-up from CheckMate 205 as well as larger randomized studies are needed for validation.

#### Combination therapy

Combination immunotherapy with nivolumab and ipilimumab, another immune checkpoint inhibitor blocking cytotoxic T-lymphocyte-associated protein 4 (CTLA-4), has been shown to be superior to monotherapies in treating some solid tumors including melanoma. Arm 2 of the CheckMate 039 study evaluated the efficacy of nivolumab and ipilimumab in hematological malignancies. Patients were treated with nivolumab (3 mg/kg) plus ipilimumab (1 mg/kg) every 3 weeks for 4 cycles, followed by nivolumab monotherapy (3 mg/kg) every 2 weeks for up to 2 years. At the time of report at ASH 2016, 65 patients were treated, including 31 HL patients. The ORR in HL patients was 74%, with 19% CR and 55% PR [60]. The CR rate appeared similar to nivolumab monotherapy (17%) [52, 56]. The benefit of adding ipilimumab in this setting needs further study.

Given the efficacy of both nivolumab and brentuximab vedotin, the combination is currently being investigated in HL. The ECOG-ACRIN E4412 trial is studying the combination of brentuximab vedotin with nivolumab or ipilimumab or both in relapsed or refractory HL. Preliminary results from arm D (nivolumab 3 mg/kg plus brentuximab vedotin 1.2 mg/kg, *n* = 3), arm E (nivolumab 3 mg/kg plus brentuximab vedotin 1.8 mg/kg, *n* = 7), and arm F (expansion cohort, *n* = 9) were reported at ICML in 2017. In 17 patients evaluable for response, the ORR was 89% and the CR rate was 50% [61]. The high CR and ORR were encouraging, suggesting a deepening of response compared with either therapy alone. In another multicenter phase 1/2 study, cHL patients who relapsed or were refractory to frontline chemotherapy were treated with nivolumab (3 mg/kg) plus brentuximab vedotin (1.8 mg/kg) every 3 weeks for up to 4 cycles. Sixty-two patients were enrolled, and 58 completed 4 cycles of treatment. In 60 patients evaluable for efficacy, the ORR was 83%, and the CR rate was 62%. Seventeen patients received additional salvage therapy subsequent to study treatment (ICE for the majority). Fifty-four patients had undergone ASCT [62]. The high CR rate in this study suggests that this combination is a very promising salvage therapy after failure to frontline chemotherapy.

### Pembrolizumab

Pembrolizumab is an IgG4 fully human anti-PD-1 mAb. Like nivolumab, it is efficacious in treating a variety of solid tumors and has also demonstrated a high activity in HL. KEYNOTE-013 was the initial phase 1b study that evaluated the safety and efficacy of pembrolizumab in patients with relapsed or refractory cHL. Thirty-one patients were enrolled. All of them had prior exposure to brentuximab vedotin, and 71% had relapsed after ASCT. Pembrolizumab (10 mg/kg) was administered every 2 weeks for 2 years or until disease progression or unacceptable toxicity. The ORR was 65%, with 16% CR and 48% PR [63]. Updated efficacy data was reported at ASH 2016 with a median follow-up of 24.9 months. Per blinded independent central review, the median PFS was 11.4 months and the 6- and 12-month PFS rates were 66 and 48%, respectively. The 6- and 12-month OS rates were 100 and 87%, respectively [64].

Following KEYNOTE-013, the phase 2 KEYNOTE-087 trial was conducted to further evaluate the efficacy of pembrolizumab in relapsed or refractory cHL. A total of 210 cHL patients who had prior ASCT and/or brentuximab vedotin were included. Pembrolizumab (200 mg) was administered every 3 weeks for 2 years or until disease progression or intolerable toxicity. The ORR by central review was 69.0%, with 22.4% CR and 46.7% PR. The response rates were similar in pre-specified cohorts (progression after [1] ASCT and subsequent brentuximab vedotin, [2] salvage chemotherapy and brentuximab vedotin, ineligible for ASCT, or [3] ASCT without post-transplant brentuximab vedotin). At 6 months, the PFS rate was 72.4% and the OS rate was 99.5%. The 9-month PFS and OS rates were 63.4 and 97.5%, respectively [65]. Based on the results of this trial, the FDA-approved pembrolizumab in March 2017 for the treatment of patients with refractory cHL, or who have relapsed after three or more prior lines of therapy.

### Avelumab

Avelumab is a fully human anti-PD-L1 mAb. Blocking the PD-1 checkpoint with avelumab has also been shown to be effective for the treatment of relapsed or refractory HL. In the phase 1 JAVELIN HOGKINS study, cHL patients who progressed after or were ineligible for stem cell transplant were treated with avelumab at five different dosing regimens (70 mg, 350 mg, 500 mg every 2 weeks (Q2W), 500 mg every 3 weeks (Q3W), or 10 mg/kg Q2W). Treatments were well tolerated. The ORR was 54.8% with two CRs (6.5%) and 15 PRs (48.4%). Responses were observed in all dosing groups, with ORR ranging from 14.3 to 83.3% [66]. The ORR appeared similar to that observed with nivolumab and pembrolizumab, but further studies are certainly needed.

## Conclusions

Classical HL has unique biology, and this provides opportunities for novel targeted therapies. Brentuximab vedotin targeting CD30 and nivolumab and pembrolizumab targeting PD-1 have demonstrated impressive single-agent activity in treating relapsed and refractory cHL and have received FDA approval for this indication. Ongoing clinical studies have been evaluating their roles in treating cHL at first relapse and at initial diagnosis and encouraging results have been demonstrated including those leading to the approval of brentuximab vedotin for untreated stage III–IV cHL patients in combination with chemotherapy. As these results impact the current standards of care, the use of these agents in earlier lines of therapy may improve the overall outcome of patients with cHL. Improving the cure rates of frontline therapy by using combinations that incorporate novel agents will not only improve disease outcome generally, but will also spare patients from long-term toxicity from conventional chemotherapy and radiotherapy. CD30- and PD-1-targeted therapies for cHL do face some challenges. In the relapsed and refractory setting, how to sequence and/or combine these agents remains unclear and requires further investigation. How to combine these agents with chemotherapy also requires further study. With a limited number of eligible patients, carrying out multiple large studies can be difficult. In the frontline setting, a clear role of these agents has yet to be established. While brentuximab vedotin plus chemotherapy is approved for untreated stage III–IV cHL, controversies do exist whether to use this combination for all patients given the small absolute benefit and high cost. The financial cost is a major challenge for all these CD30- and PD-1-targeted therapies in general.

## Abbreviations

ABVD: Adriamycin, bleomycin, vinblastine, dacarbazine
ACRIN: American College of Radiology Imaging Network
ADC: Antibody-drug conjugate
AE: Adverse event
ALCL: Anaplastic large-cell lymphoma
ALL: Acute lymphoblastic leukemia
Allo-SCT: Allogeneic stem cell transplantation
ASCT: Autologous stem cell transplantation
ASH: American Society of Hematology
AVD: Adriamycin, vinblastine, dacarbazine
BEACOPP: Bleomycin, etoposide, doxorubicin, cyclophosphamide, vincristine, procarbazine, prednisone
BrECADD: Brentuximab vedotin, etoposide, cyclophosphamide, doxorubicin, dacarbazine, and dexamethasone
BrECAPP: Brentuximab vedotin, etoposide, cyclophosphamide, doxorubicin, procarbazine, and prednisone
CAR: Chimeric antigen receptor
CD: Cluster of differentiation
cHL: Classical Hodgkin lymphoma
CMR: Complete metabolic response
CR: Complete response
CTLA-4: Cytotoxic T-lymphocyte-associated protein 4
DOR: Duration of response
ECADD: Etoposide, cyclophosphamide, doxorubicin, dacarbazine, and dexamethasone
ECAPP: Etoposide, cyclophosphamide, doxorubicin, procarbazine, and prednisone
ECOG: Eastern Cooperative Oncology Group
EFS: Event-free survival
EORTC: European Organisation for Research and Treatment of Cancer
ESHAP: Etoposide, Solu-Medrol, high-dose cytarabine, cisplatin
FDA: Food and drug administration
FFS: Failure-free survival
FIL: Fondazione Italiana Linfomi
GELTAMO: Grupo Español de linfomas/trasplante autólogo de médula ósea
GM-CSF: Granulocyte-macrophage colony-stimulating factor
GVHD: Graft-versus-host disease
HDAC: Histone acetyltransferase
HDT: High-dose therapy
HL: Hodgkin lymphoma
HR: Hazard ratio
HRS: Hodgkin and Reed-Sternberg
ICE: Ifosfamide, carboplatin, etoposide
ICML: International Conference on Malignant Lymphoma
IFRT: Involved-field radiotherapy
IMiD: Immunomodulatory drug
INRT: Involved-node radiotherapy
ISRT: Involved-site radiotherapy
JAK: Janus kinase
Lysa: Lymphoma study association
mAb: Monoclonal antibody
MMAE: Monomethyl auristatin E
MR: Minor response
MTD: Maximum tolerated dose
mTOR: Mammalian target of rapamycin
NCCN: National Comprehensive Cancer Network
NLPHL: Nodular lymphocyte predominant Hodgkin lymphoma
ORR: Objective response rate
OS: Overall survival
PD: Progressive disease
PD-1: Programmed cell death protein 1
PD-L1: Programmed death-ligand 1
PD-L2: Programmed death-ligand 2
PET: Positron emission tomography
PFS: Progression-free survival
PI3K: Phosphatidyl-inositide 3 kinase
PR: Partial response
Q2W: Every 2 weeks
Q3W: Every 3 weeks
SD: Stable disease
STAT: Signal transducers and activators of transcription
TNF: Tumor necrosis factor
